# Circulating ceramide ratios and risk of vascular brain aging and dementia

**DOI:** 10.1002/acn3.50973

**Published:** 2020-01-16

**Authors:** Emer R. McGrath, Jayandra J. Himali, Vanessa Xanthakis, Meredith S. Duncan, Jean E. Schaffer, Daniel S. Ory, Linda R. Peterson, Charles DeCarli, Matthew P. Pase, Claudia L. Satizabal, Ramachandran S. Vasan, Alexa S. Beiser, Sudha Seshadri

**Affiliations:** ^1^ Department of Neurology Brigham & Women’s Hospital Boston Massachusetts; ^2^ Harvard Medical School Boston Massachusetts; ^3^ Framingham Heart Study Framingham Massachusetts; ^4^ School of Public Health Boston University Boston Massachusetts; ^5^ Boston University School of Medicine Boston Massachusetts; ^6^ Glenn Biggs Institute for Alzheimer’s & Neurodegenerative Diseases University of Texas Health Sciences Center San Antonio Texas; ^7^ Vanderbilt University Medical Center Nashville Tennessee; ^8^ Washington University School of Medicine St Louis Missouri; ^9^ Department of Neurology University of California Davis California; ^10^ Melbourne Dementia Research Centre The Florey Institute for Neuroscience and Mental Health Victoria Australia; ^11^ The University of Melbourne Victoria Australia

## Abstract

**Background:**

We determined the association between ratios of plasma ceramide species of differing fatty‐acyl chain lengths and incident dementia and Alzheimer’s disease (AD) dementia in a large, community‐based sample.

**Methods:**

We measured plasma ceramide levels in 1892 [54% women, mean age 70.1 (SD 6.9) yr.] dementia‐free Framingham Offspring Study cohort participants between 2005 and 2008. We related ratios of very long‐chain (C24:0, C22:0) to long‐chain (C16:0) ceramides to subsequent risk of incident dementia and AD dementia. Structural MRI brain measures were included as secondary outcomes.

**Results:**

During a median 6.5 year follow‐up, 81 participants developed dementia, of whom 60 were diagnosed with AD dementia. In multivariable Cox‐proportional hazards analyses, each standard deviation (SD) increment in the ratio of ceramides C24:0/C16:0 was associated with a 27% reduction in the risk of dementia (HR 0.73, 95% CI 0.56–0.96) and AD dementia (HR 0.73, 95% CI 0.53–1.00). The ratio of ceramides C22:0/C16:0 was also inversely associated with incident dementia (HR per SD 0.75, 95% CI 0.57–0.98), and approached statistical significance for AD (HR 0.73, 95% CI 0.53–1.01, *P* = 0.056). Higher ratios of ceramides C24:0/C16:0 and C22:0/C16:0 were also cross‐sectionally associated with lower white matter hyperintensity burden on MRI (−0.05 ± 0.02, *P* = 0.02; −0.06 ± 0.02, *P* = 0.003; respectively per SD increase), but not with other MRI brain measures.

**Conclusions:**

Higher plasma ratios of very long‐chain to long‐chain ceramides are associated with a reduced risk of incident dementia and AD dementia in our community‐based sample. Circulating ceramide ratios may serve as potential biomarkers for predicting dementia risk in cognitively healthy adults.

## Introduction

Discovering novel blood‐based biomarkers for dementia can identify disease at an early preclinical stage, serve as surrogate outcomes for clinical trials of investigational therapies and inform our understanding of the underlying biological pathways of disease onset and progression. Altered lipid metabolism is believed to play an important role in the development of dementia and Alzheimer’s disease (AD).^1^, ^2^, ^3^ Ceramides are molecular species belonging to the sphingolipid family, consisting of a sphingoid base coupled with a fatty acyl chain, differing from each other according to the length of the fatty acyl chain.^4^ They are generated through three pathways, that is, the cleavage of sphingomyelin by sphingomyelinases, the salvage of breakdown products of complex sphingolipids, and de novo synthesis via a family of six ceramide synthase enzymes.^5^ Ceramides play roles in low density lipoprotein aggregation,^6^ inflammation,^7^ endothelial dysfunction,^8^ and cell proliferation, differentiation and apoptosis, including neuronal cell death.^9^, ^10^, ^11^ Total plasma ceramide concentrations have been suggested as potential early blood‐based biomarkers for predicting mild cognitive impairment and progression to dementia.^12^ Prior studies have reported an association between higher plasma ceramide concentrations and hippocampal atrophy,^1^ cognitive decline,^13^ as well as all‐cause dementia and AD dementia, although results have been conflicting between men and women.^14^, ^15^ Additionally, many of these studies were limited by smaller sample sizes and a cross‐sectional design.

Recently, attention has focused on the role of circulating ratios of a very‐long‐chain to long‐chain ceramides as biomarkers for major vascular events^16^, ^17^ and the possibility that these ratios may be of greater diagnostic and prognostic value for cardiovascular outcomes compared to total ceramide levels or concentrations of individual ceramide species. It has been contended, but is unproven, that the adverse cognitive effects of ceramides may be related to the relative proportions of circulating very long‐chain to long‐chain fatty acyl chains rather than simply due to elevated total ceramide levels.^18^, ^19^ In the present investigation, we related circulating ratios of previously measured very long‐chain to long‐chain ceramides^17^ to MRI structural brain measures (cross‐sectionally) and amyloid burden on brain‐PET (longitudinally), and to the incidence of clinically confirmed dementia and AD dementia prospectively in a large, community‐based, sample of relatively healthy adults.

## Methods

### Study sample

**Figure 1:**
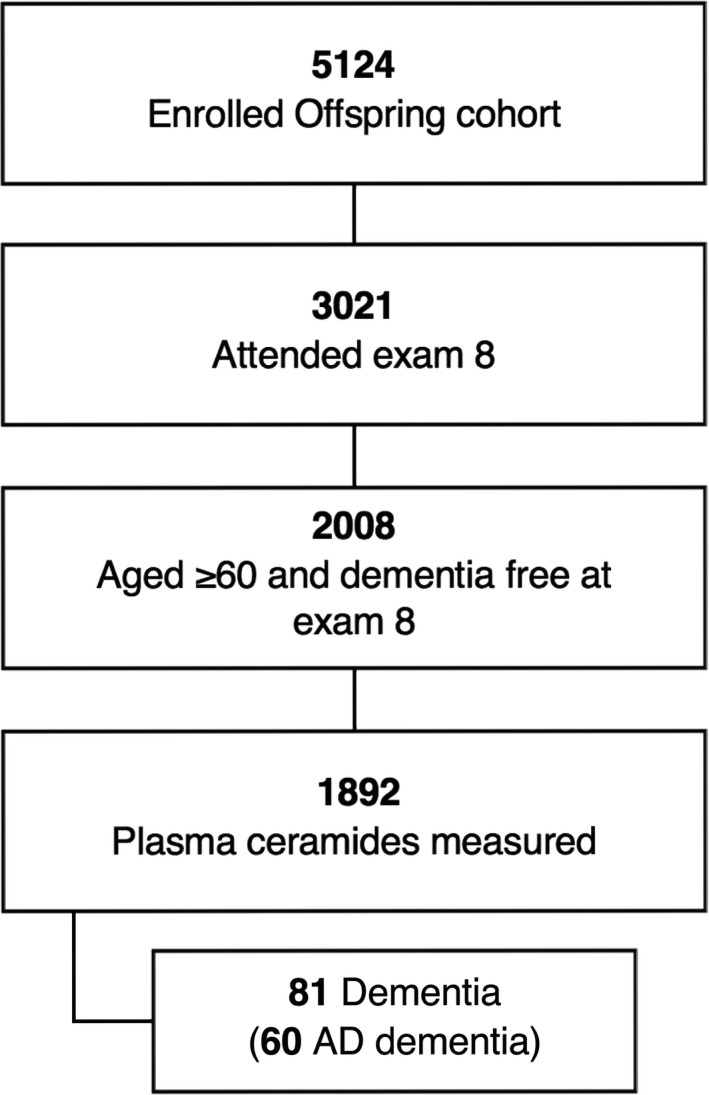
Sample selection for present investigation.

The Framingham Offspring cohort is a longitudinal epidemiological study of individuals recruited between 1971 and 1975. This large, predominantly middle‐aged cohort has been prospectively followed for the development of vascular risk factors, decline in cognitive function, dementia and stroke for over 45 years.^20^ Each participant is evaluated at approximately 4‐year intervals from the time of enrollment into the cohort. In the present investigation, we included participants from the Offspring cohort who attended their eight quadrennial examination cycle (2005–2008), were aged 60 years or above and free of dementia at this exam, and who had data available on dementia status at follow‐up and had undergone plasma ceramide measurements at that examination. All participants provided written informed consent. The institutional review board at Boston University Medical Center approved the study protocol. Of the original Offspring cohort (*n* = 5124), 3021 were alive and attended examination eight, 2008 of whom were aged 60 years or above, confirmed free of dementia and with available dementia status on follow‐up. Of these, 1892 had plasma ceramides measured at examination eight, resulting in the sample for the present investigation (Fig. 1). For the cross‐sectional analyses of structural MRI brain measures, we included those participants in the dementia cohort who had an MRI brain completed at exam eight (*n* = 1541 [81% of the dementia cohort]) and had available plasma ceramide measurements. For analyses of amyloid‐burden on brain‐PET, we included participants in the dementia cohort who had available PET data at exam nine (*n* = 48).

### Outcome measures

Our primary outcome measure was incident all‐cause dementia developing at any time after the 8th examination cycle and up to the end of 2016. From examination cycle five (1991–1995) onward, all participants in the Offspring cohort were systematically screened using the Mini‐Mental State Examination (MMSE) and annual health status updates for the development of dementia. From examination cycle seven onward, we invited all participants to complete neurocognitive testing. We completed more detailed cognitive testing in those cases where the participant’s MMSE score was lower than the education‐based cut‐off, three points lower than the preceding examination, or five points lower than the previous highest recorded score or if the participant, family member, or Framingham study physician was concerned about cognitive impairment.^21^ If a participant had suspected cognitive impairment but did not meet criteria for a diagnosis of dementia, additional yearly neuropsychological assessments were performed between the quadrennial Offspring examinations. A diagnosis of dementia was based on the Diagnostic and Statistical Manual of Mental Disorders (4th edition) criteria requiring impairment in memory and at least one other domain of cognitive function, along with impaired functional ability. Adjudication of dementia, and date of diagnosis, was reached by a committee comprising of at least one neuropsychologist and one neurologist after a detailed review of all available neurological examination records, neuropsychological assessments, neuro‐imaging data, hospital/nursing home/outpatient clinic records, information from family interviews, and autopsy results (when available). AD dementia was included as an additional primary outcome measure. A diagnosis of AD dementia was reached based on the criteria of the National Institute of Neurological and Communicative Disorders and Stroke and the Alzheimer’s Disease and Related Disorders Association for definite, probable, or possible Alzheimer’s disease.^22^

We evaluated structural MRI brain measures in secondary analyses, including total parenchymal brain volume (TBV), hippocampal volume (HV), white matter hyperintensity volume (WMHV) and covert brain infarcts (CBI). MRI imaging was performed using a Siemens Avanto 1.5 Tesla machine. More detailed methods on MRI brain measures have previously been published.^23^ In brief, TBV was defined as supratentorial brain volume as a percentage of the intracranial volume, determined from coronal sections. White matter hyperintensity volume (WMHV) was performed on a combination of FLAIR and 3D T1 images using a modified Bayesian probability structure based on a previously published method of histogram fitting.^24^ Cerebral volumes were calculated as a percentage of intracranial volume and then natural logarithmically transformed to approximate a normal distribution. Hippocampal volume (also calculated as a percentage of intracranial volume) was measured using an automatic hippocampal segmentation method employing a standard atlas‐based diffeomorphic approach,^25^ which was modified to include the European Alzheimer's Disease Consortium‐Alzheimer's Disease Neuroimaging Initiative (EADC‐ADNI) harmonized hippocampal masks. The presence of CBI was manually determined based on the size (≥3 mm), location and imaging characteristics of the lesion according to the STandards for ReportIng Vascular changes on nEuroimaging (STRIVE) criteria,^26^ with documented good interrater reliability.^27^, ^28^ Imaging data were centrally processed at the Imaging of Dementia and Aging (IDeA) laboratory located at UC Davis and analyzed by operators blinded to all participant characteristics including ceramide levels. We included amyloid‐PET burden using ^11^‐C PiB as a secondary outcome measure in exploratory analyses (see Appendix for further details).

### Measurement of plasma ceramides

Fasting blood samples were drawn from participants in the early morning, centrifuged and stored at −80°C until assays were performed. A modified and fully validated two‐dimensional liquid chromatography‐tandem mass spectrometry (LC–MS) assay was used to quantify plasma levels of ceramides C24:0, C22:0, and C16:0 according to previous published methods.^17^ Linear dynamic ranges for ceramides C16:0, C22:0, and C24:0 in this assay were 0.01 to 2, 0.04 to 8, and 0.1 to 20 µg/mL, respectively. The intraassay and interassay precisions were within 6.9%, 7.6% and 7.8% coefficient of variation for C24:0, C22:0 and C16:0, respectively.^17^

### Covariates

We adjusted for baseline demographics and covariates (measured at examination cycle eight), which have previously been associated with plasma ceramide ratios and risk of dementia^17^, ^29^ (variables were selected based on clinical importance and evidence of prior associations), including age, sex, education (self‐reported and categorized as: no high school degree, high school degree but no college degree, some college but no degree, college degree or higher), systolic blood pressure/use of antihypertensive medication, apolipoprotein E4 (ApoE4) carrier status (a carrier was defined as E2/E4, E3/E4 or E4/E4; a noncarrier was defined as E2/E2, E2/E3 or E3/E3), prevalent cardiovascular disease (CVD, which included peripheral vascular disease [including intermittent claudication]; coronary artery disease [including coronary insufficiency, angina, myocardial infarction]; and cerebrovascular disease [including transient ischemic attack and stroke]; and congestive heart failure]), ratio of total cholesterol to high‐density lipoprotein cholesterol (TC:HDL‐C), serum triglycerides, and use of lipid‐lowering therapies.

### Statistical analysis

We standardized ceramide values to facilitate comparisons as continuous variables. Multivariable Cox proportional hazards models were estimated to evaluate the association between circulating ceramide ratios and risk of incident all‐cause dementia and AD dementia. Participants were followed from the baseline examination cycle eight to the time of the incident event. For individuals without incident events, follow‐up was censored at the time of death or the date the participant was last known to be dementia free, through December 2016. The assumption of proportionality of hazards was confirmed, and results are reported as hazard ratios (HR) with corresponding 95% confidence intervals (CI). Model one adjusted for age and sex, and model two additionally adjusted for education, systolic blood pressure, use of antihypertensive medication, prevalent CVD, ApoE4 carrier status; TC:HDL ratio, use of lipid‐lowering therapies, and serum TG.

We estimated logistic (for binary outcomes) and linear (for continuous measures) regression models to evaluate the cross‐sectional associations between circulating ceramide ratios and MRI‐based structural brain measures, including CBI, WMHV, TBV, and HV. Models were adjusted for age, age squared (given age and brain volume show a nonlinear association), sex, time from blood draw to MRI brain, use of antihypertensive medication, systolic blood pressure, prevalent cardiovascular disease, TC:HDL ratio, use of lipid‐lowering therapies, and serum TG. We investigated for interactions between ceramide ratios and sex, ApoE4 carrier status and TC:HDL‐C ratio (<4:1 vs. ≥4:1) for the primary outcome of incident dementia*.* We also completed a sensitivity analysis excluding those with a history of prior stroke (*n* = 52). Our primary, a priori, analyses evaluated the associations between the ratio of ceramides C24:0 to C16:0 and C22:0 to C16:0 and risk of incident dementia. In secondary (exploratory) analyses, we related plasma concentrations of individual ceramide species (i.e., C16:0, C22:0 and C24:0) to the risk of incident dementia and AD dementia prospectively, and with structural MRI brain measures, cross‐sectionally. In addition, we estimated multivariable linear regression models to relate plasma ceramide ratios and species to amyloid‐PET burden, adjusting for age, sex, and time from blood draw to PET scan. A two‐sided *P* < 0.05 was considered statistically significant. We completed all analyses using SAS version 9.4 (SAS Institute Inc., Cary, NC).

## Results

Our sample included 1892 eligible participants. The mean age of the cohort was 70.1 (SD 6.9) years and 54% were women. Baseline characteristics are shown in Table 1.

**Table 1:** Baseline characteristics.

Variable	Overall (*n* = 1892)
No. (%)	
Age, y, mean (SD)	70.1 (6.9)
Women	1022 (54.0)
Systolic blood pressure, mmHg, mean (SD)	130.3 (17.1)
Ceramide 16:0, μg/mL (Q1, Q3)	0.16 (0.14, 0.19)
Ceramide 22:0, μg/mL (Q1, Q3)	0.59 (0.49, 0.71)
Ceramide 24:0, μg/mL (Q1, Q3)	2.17 (1.81, 2.60)
Ceramide 24:0/16:0	13.43 (11.64–15.67)
Ceramide 22:0/16:0	3.65 (3.16–4.21)
TC, mg/dL, mean (SD)	183.3 (37.0)
HDL, mg/dL, mean (SD)	57.1 (18.2)
TC:HDL, mean (SD)	3.44 (1.04)
TG, mg/dL, mean (SD)	117.7 (68.2)
Education	
No high school degree	80 (4.3)
High school degree	553 (29.6)
Some years of college	567 (30.4)
College degree	668 (35.8)
Anti‐hypertensive medication	1022 (54.1)
Lipid lowering therapy	892 (47.2)
ApoE4 allele carrier	398 (21.7)
Prevalent CVD	351 (18.6)
Prior stroke	52 (2.7%)

Baseline demographic and clinical characteristics were defined at examination 8.

Abbreviations: SD, standard deviation; CVD, cardiovascular disease; APOE E4, apolipoprotein E4 allele carrier (defined as E2/E4, E3/E4 or E4/E4).

### Ceramide ratios and incident dementia

During a median follow up of 6.5 (IQR 5.5–7.7) years, 81 individuals were diagnosed with dementia, 60 of whom had AD dementia. In multivariable Cox‐proportional hazards regression models adjusted for demographics, vascular risk factors, use of lipid‐lowering therapies and ApoE4 carrier status, the ratio of ceramides C24:0/C16:0 was inversely associated with incident dementia (Hazards ratio [HR] per each standard deviation [SD] increment 0.73, 95% CI 0.56–0.96, *P* = 0.023) and AD dementia (HR 0.73, 95% CI 0.53–1.00, *P* = 0.050). The ratio of ceramides C22:0/C16:0 was also inversely associated with incident dementia (HR per SD 0.75, 95% CI 0.57–0.98, *P* = 0.038), but not AD dementia (HR per SD 0.73, 95% CI 0.53–1.01, *P* = 0.056) (Table 2). A sensitivity analysis excluding those with prior stroke showed results consistent with our primary analyses. There was no statistically significant interaction according to sex, ApoE4 carrier status or TC:HDL‐C ratio for the risk of dementia associated with any of the ceramide levels or ratios.

**Table 2:** Ceramide ratios and risk of incident dementia and AD dementia.

	All‐cause dementia				Alzheimer’s disease dementia			
	Model 1		Model 2		Model 1		Model 2	
	HR (95% CI)	*P*‐value	HR (95% CI)	*P*‐value	HR (95% CI)	*P*‐value	HR (95% CI)	*P*‐value
Ceramide 24:0/16:0	0.76 (0.59–0.98)	0.03	0.73 (0.56–0.96)	0.023	0.77 (0.57–1.04)	0.08	0.73 (0.53–1.00)	0.050
Ceramide 22:0/16:0	0.80 (0.62–1.03)	0.08	0.75 (0.57–0.98)	0.038	0.80 (0.59–1.07)	0.13	0.73 (0.53–1.01)	0.056

HR is reported per standard deviation unit increment in ceramide ratio.

Model 1: adjusted for age and sex.

Model 2: adjusted for age, sex, education, systolic blood pressure, use of antihypertensive medication, prevalent cardiovascular disease, and ApoE4 carrier status, TC:HDL ratio, use of lipid‐lowering therapies and serum TG.

### Ceramide ratios and MRI and PET brain measures

In multivariable analyses adjusting for age, age squared, sex, time from blood draw to MRI brain, systolic blood pressure, use of antihypertensive medication, prevalent cardiovascular disease, TC:HDL ratio, use of lipid‐lowering therapies, and serum TG, each standard deviation increment in the ratio of ceramides C24:0/C16:0 and C22:0/C16:0 was associated with lower WMHV (*β* ± SE, −0.05 ± 0.02, *P* = 0.02; −0.06 ± 0.02, *P* = 0.003; respectively; Table 3). Ceramide ratios were not cross‐sectionally associated with hippocampal volume, total brain volume or with number of covert brain infarcts on brain MRI. In an exploratory analysis among 48 individuals with available PiB‐PET data, each SDU increment in the ratio of ceramide 24:0/16:0 was associated with a reduced burden of ß‐amyloid on PET (0.12 ± 0.06, *P* = 0.05) (Table S1).

**Table 3:** Ceramide ratios and MRI markers of structural brain injury.

	TBV		Hippocampal volume		WMHV^a^		Covert brain infarcts	
	*β* ± SE	*P*‐value	*β* ± SE	*P*‐value	*β* ± SE	*P*‐value	OR (95% CI)	*P*‐value
Ceramide 24:0/16:0	0.03 ± 0.05	0.44	−0.001 ± 0.001	0.46	−0.05 ± 0.02	0.02	0.98 (0.82–1.17)	0.79
Ceramide 22:0/16:0	0.07 ± 0.05	0.17	−0.0004 ± 0.001	0.74	−0.06 ± 0.02	0.003	1.04 (0.87–1.24)	0.68

Model adjusted for age, age squared, sex, time from blood draw to MRI brain, systolic blood pressure, use of antihypertensive medication, prevalent cardiovascular disease, TC:HDL ratio, use of lipid‐lowering therapies, and serum TG.

Abbreviations: SDU, Standard deviation units; SE, Standard error; TBV, total brain volume; WMHV, White matter hyperintensity volume.

a Natural log transformed.

### Secondary analyses ‐ individual ceramide species

In secondary analyses of individual ceramide species, higher concentrations of ceramide C16:0 were also independently associated with an increased risk of AD dementia (HR per SD 1.49, 95% CI 1.08–2.06, *P* = 0.01) but not overall dementia (HR per SD 1.21, 95% CI 0.92–1.61, *P* = 0.18). None of the other ceramide species was individually associated with dementia risk (Table S2). Higher plasma ceramide C16:0 concentrations were also cross‐sectionally associated with a greater volume of white matter hyperintensities on MRI brain (per SD increment, ß ± SE, 0.05 ± 0.03, *P* = 0.04, Table S3). None of the individual plasma ceramides species was independently associated with ß‐amyloid burden on brain PET (Table S1).

## Discussion

In the present investigation, higher ratios of very long‐chain to long‐chain ceramides were associated with a lower risk of dementia (C24:0/C16:0 and C22:0/C16:0) and AD dementia (C24:0/C16:0) prospectively, as well as with a lower burden of white matter disease on MRI brain and B‐amyloid burden on PET (C24:0/C16:0).

Previous studies have reported conflicting findings regarding the association between individual plasma ceramide species and the risk of AD dementia. Some studies have reported an association of both elevated long‐chain ceramides (C16:0)^1^, ^14^, ^15^ and very long‐chain ceramides (C22:0 and C24:0)^14^, ^15^ with the risk of dementia or AD dementia, whereas others observed no association of very long‐chain ceramides^1^ with dementia risk. Some of these studies were limited by their cross‐sectional design or small sample sizes. In addition, results have varied across tertiles of ceramide levels and according to sex, with positive associations noted in women in one study,^14^ but only in men in a second study.^15^ To our knowledge, there have been no prior studies to date exploring the relationship between ratios of ceramides of varying fatty acyl chain length and the subsequent risk of dementia. For each standard deviation, increment in the ratio of ceramides C24:0 to C16:0 and C22:0 to C:16:0, there was an approximate 25% lower risk of developing dementia in our investigation. We did not observe an interaction according to sex for the risk of all‐cause dementia associated with any of the ceramide levels or ratios.

Individual ceramide species are believed to have different functions according to their length of fatty acyl chain. While very long‐chain fatty acyl ceramides are important for myelin function and have been suggested to protect against dementia,^18^ long‐chain ceramide species have been linked with deleterious proinflammatory and apoptotic effects.^11^, ^18^, ^30^ Abnormalities in the myelin sphingolipid pathway, including the synthesis of very long‐chain ceramides, have been shown to occur in preclinical and clinical stages of AD dementia. Reduced ceramide synthase‐2 activity (the enzyme responsible for catalyzing the production of very long‐chain ceramides), has been found in early Braak stages I/II in the temporal cortex and in stages III/IV in the frontal cortex and hippocampus of human brain tissue.^19^ In addition, severe depletion of sulfatide and galactosylceramide, and their direct precursor, very long‐chain ceramides, has also been found in AD brain tissue, along with a corresponding increase in the proportion of long‐chain ceramide species.^19^ Our findings of an increased risk of dementia in those with lower levels of circulating very long‐chain ceramides relative to long‐chain ceramides are consistent with these findings and may suggest a deleterious effect of long‐chain ceramides and an apparent protective effect of very long‐chain ceramides on the risk of dementia. If confirmed, our findings raise the possibility that circulating ceramide ratios may be useful predictors of adverse cognitive outcomes and may offer predictive utility in the early or even preclinical stages of dementia.

Recent cardiovascular studies have consistently shown an increased risk of adverse vascular events and mortality in individuals with increased ratios of long‐chain to very long‐chain ceramides in plasma.^5^, ^17^, ^31^, ^32^, ^33^ In a murine model of ceramide synthase 2 haploinsufficiency, a reduction in levels of very long‐chain (C22:0 and C24:0) ceramides and a compensatory increase in levels of long‐chain ceramides (C16:0) was noted, resulting in an increased susceptibility to insulin resistance and hepatocyte apoptosis.^30^ Given the role of ceramides in pancreatic *β*‐cell dysfunction, insulin resistance and vascular reactivity,^34^ increased cardiometabolic risk could potentially mediate the association with dementia. Indeed, we observed that higher levels of ceramides C24:0 and C22:0 relative to C16:0 were associated with a lower burden of white matter disease on MRI brain. Given that interventions lowering total ceramide levels have been shown to prevent atherosclerosis and insulin resistance in murine models,^35^, ^36^ modification of ceramides could represent one attractive therapeutic option for prevention of vascular contributions to dementia, a premise that remains to be tested.

In a small exploratory analysis within our cohort (*n* = 48), an elevated ratio of C24:0 to C16:0 was associated with reduced ß‐amyloid burden on PET‐brain. Ceramides have been shown to stimulate ß‐amyloid formation, the pathological hallmark of AD, through regulating ß‐site amyloid precursor protein cleaving enzyme 1 (BACE‐1) activity,^37^, ^38^ while inhibition of ceramide synthesis results in reduced production of ß‐amyloid.^37^, ^38^, ^39^, ^40^ There is also evidence to support a role for ceramides in tau phosphorylation. The enzyme protein phosphatase 2A (PP2A), the primary regulator of tau phosphorylation in the brain, appears to be modulated by long‐chain ceramides such as C18:0.^41^, ^42^ In a murine model, inhibition of serine palmitoyltransferase (a rate‐limiting enzyme in de novo ceramide synthesis) resulted in downregulation of both cortical ß‐amyloid and hyperphosphorylated tau levels.^43^ Thus, it is conceivable that pharmacological inhibition of long‐chain ceramide synthesis could also slow down or prevent the progression of AD dementia through the prevention of ß‐amyloid accumulation and tau phosphorylation.

Important strengths of our study include the use of a community‐based sample of cognitively healthy adults confirmed to be free of dementia at baseline, a rigorous and standardized approach to diagnosis of clinical dementia and AD along with in‐depth surveillance procedures, a careful assessment of risk factors, and the large number of individuals with measured plasma ceramide concentrations compared to other studies to date. Furthermore, the assay used to measure plasma ceramides has shown high precision and accuracy with minimal interbatch variability. Notable limitations include the short follow‐up interval (median 6‐year follow up) with a modest number of outcome events and the need for validation of these findings in other cohorts. Furthermore, blood ceramide concentrations may change over time and we were unable to assess stability of the ratios during follow‐up via repeat measurements. In addition, the Framingham Offspring Study is predominantly white and this will limit the generalizability of our findings to other races/ethnicities.

## Conclusions

To our knowledge, this is the first study to demonstrate that higher ratios of very long‐chain to long‐chain ceramides in the blood are associated with a reduced risk of incident dementia and AD dementia. Our study provides evidence supporting the hypothesis that individual ceramide species have differing biological roles in the pathophysiology of dementia. If our findings are confirmed, circulating ratios of very long‐chain to long‐chain ceramides may be useful biomarkers for predicting future dementia risk among cognitively healthy adults, several years in advance of clinically evident disease.

## Author Contributions

Study concept and design: McGrath, Seshadri, Himali, Vasan. Acquisition, analysis, or interpretation of data: All authors. Drafting of the manuscript: McGrath. Critical revision of the manuscript for important intellectual content: All authors. Statistical analysis: Himali. Obtained funding: Seshadri, McGrath. Study supervision: Seshadri.

## Conflicts of Interest

None to report.

## Supporting information


**Data S1.** Supplementary methods.
**Table S1.** Ceramides and amyloid burden on PET.
**Table S2.** Individual ceramide species and risk of incident dementia and AD dementia.
**Table S3.** Individual ceramide species and MRI markers of structural brain injury
