# Supplementary material for: Circulating ceramide ratios and risk of vascular brain aging and dementia
Source: Ann Clin Transl Neurol. 2020 Jan 16;7(2):160–8. doi: 10.1002/acn3.50973 (PMC7034495; doi:10.1002/acn3.50973)
Supplement: Supplementary file 1 — Data S1. Supplementary methods. Table S1. Ceramides and amyloid burden on PET. Table S2. Individual ceramide species and risk of incident dementia and AD dementia. Table S3. Individual ceramide species and MRI markers of structural brain injury [file ACN3-7-160-s001.docx]

**Supplementary appendix**

Supplementary methods

Amyloid-PET burden was measured using ^11^-C PiB. MRI brain studies were first completed using a 3-Tesla Phillips Achieva scanner, followed by acquisition of PET images using a Siemens/CTI ECAT HR+ scanner. The PET acquisition and processing protocols have previously been described^1^ and are validated against tissue amyloid burden at autopsy.^2^ In brief, a transmission scan is obtained to correct for attenuation, followed by injection of ^11^-C PiB and imaging acquisition from 0–60 minutes after injection. All PET data were reconstructed, and scatter and attenuation corrected, using commercially available routines. Images were inspected for adequate count statistics and head motion between frames, and corrected as needed. High resolution 3T MP-RAGE MRI images were processed prior to the PET scanning (semi-automatic, Freesurfer segmentation algorithm^3^ and a Freesurfer template atlas.^4^ For each study participant, the PiB image was aligned to that individual’s MP-RAGE MR data to yield the co-registration transformation in the native space of the MR data, using Statistical Parametric Mapping (SPM2; Wellcome Department of Cognitive Neurology). In addition, each subject’s PiB PET data were spatially normalized to a template brain, based on pixel intensity basis vectors, and smoothed with a Gaussian filter (e.g., 8-mm FWHM), using SPM.^1^

Distribution volume ratios (DVR) were calculated for each region of interest (ROI) or voxel based on the Logan graphical analysis technique^5-7^ that has been validated for PiB imaging.^8^ Time-activity curves were generated in each brain region (ROI or voxel) and in a reference region in cerebellar cortex known to have negligible specific binding of PiB (cerebellar cortex has minimal fibrillar amyloid binding).^9^ ^11^-C PiB retention was assessed as a large aggregate cortical ROI consisting of frontal, lateral temporal and parietal and retrosplenial cortices (the FLR region). We used partial volume corrected values, to account for brain atrophy.^1^ There is substantial uptake of PiB in the FLR region of patients with diagnosed AD,^10^ which has been used as a summary measure of global B-amyloid retention in multiple prior studies.^11,12^

Supplementary tables

**eTable 1. Ceramides and amyloid burden on PET**

|  | **Global ß-amyloid DVR** | |
| --- | --- | --- |
|  | **β±SE** | **P-value** |
| **Ceramide 24:0/16:0** | -0.12±0.06 | 0.05 |
| **Ceramide 22:0/16:0** | -0.09±0.07 | 0.17 |
| **Ceramide 16:0** | 0.07±0.07 | 0.33 |
| **Ceramide 22:0** | -0.02±0.07 | 0.78 |
| **Ceramide 24:0** | -0.06±0.07 | 0.40 |

Abbreviations: DVR, distribution volume ratio.

B-amyloid burden was assessed as a large aggregate of cortical regions of interest consisting of frontal, lateral temporal and parietal and retrosplenial cortices (the FLR region). Data were available on PET-amyloid for 48 individuals with plasma ceramides.

Model adjusted for age, sex and time from blood draw to PET scan.

**eTable 2. Individual ceramide species and risk of incident dementia and AD dementia**

|  | **All-cause dementia** | | | | **Alzheimer’s disease dementia** | | | |
| --- | --- | --- | --- | --- | --- | --- | --- | --- |
|  | **Model 1** | | **Model 2** | | **Model 1** | | **Model 2** | |
|  | **HR (95% CI)** | **P-value** | **HR (95% CI)** | **P-value** | **HR (95% CI)** | **P-value** | **HR (95% CI)** | **P-value** |
| **Ceramide 16:0** | 1.16 (0.92-1.45) | 0.20 | 1.21 (0.92-1.61) | 0.18 | 1.25 (0.97-1.62) | 0.08 | 1.49 (1.08-2.06) | 0.01 |
| **Ceramide 22:0** | 0.96 (0.76-1.21) | 0.72 | 0.89 (0.66-1.20) | 0.43 | 1.01 (0.78-1.30) | 0.95 | 1.01 (0.72-1.42) | 0.95 |
| **Ceramide 24:0** | 0.91 (0.72-1.15) | 0.45 | 0.84 (0.63-1.11) | 0.21 | 0.97 (0.75-1.26) | 0.82 | 0.95 (0.69-1.31) | 0.73 |

Model 1: adjusted for age and sex.

Model 2: adjusted for age, sex, education, systolic blood pressure, use of antihypertensive medication, prevalent cardiovascular disease and ApoE4 carrier status, TC:HDL ratio, use of lipid-lowering therapies and serum TG

**eTable 3. Individual ceramide species and MRI markers of structural brain injury**

|  | **TBV** | | **Hippocampal volume** | | **WMHV*** | | **Covert brain infarcts** | |
| --- | --- | --- | --- | --- | --- | --- | --- | --- |
|  | **β±SE** | **p-value** | **β±SE** | **p-value** | **β±SE** | **p-value** | **OR (95% CI)** | **p-value** |
| **Ceramide 16:0** | -0.10±0.05 | 0.08 | 0.001±0.001 | 0.49 | 0.05±0.03 | 0.04 | 1.17 (0.96-1.42) | 0.12 |
| **Ceramide 22:0** | -0.02±0.06 | 0.74 | 0.0001±0.001 | 0.92 | -0.02±0.02 | 0.42 | 1.16 (0.95-1.41) | 0.15 |
| **Ceramide 24:0** | -0.03±0.05 | 0.56 | -0.0002±0.001 | 0.88 | -0.01±0.02 | 0.72 | 1.07 (0.88-1.29) | 0.50 |

Abbreviations: SDU, Standard deviation units; SE, Standard error; TBV, total brain volume; WMHV, White matter hyperintensity volume.

Model adjusted for age, age squared, sex, time from blood draw to MRI brain, systolic blood pressure, use of antihypertensive medication, prevalent cardiovascular disease, TC:HDL ratio, use of lipid-lowering therapies, and serum TG.

*Natural log transformed

**Supplementary references**

**1.** Becker JA, Hedden T, Carmasin J, et al. Amyloid-β associated cortical thinning in clinically normal elderly. *Annals of neurology.* 2011;69(6):1032-1042.

**2.** Ikonomovic MD, Klunk WE, Abrahamson EE, et al. Post-mortem correlates of in vivo PiB-PET amyloid imaging in a typical case of Alzheimer's disease. *Brain.* 2008;131(Pt 6):1630-1645.

**3.** Fischl B, Salat DH, Busa E, et al. Whole brain segmentation: automated labeling of neuroanatomical structures in the human brain. *Neuron.* 2002;33(3):341-355.

**4.** Desikan RS, Segonne F, Fischl B, et al. An automated labeling system for subdividing the human cerebral cortex on MRI scans into gyral based regions of interest. *Neuroimage.* 2006;31(3):968-980.

**5.** Logan J, Fowler JS, Volkow ND, Wang GJ, Ding YS, Alexoff DL. Distribution volume ratios without blood sampling from graphical analysis of PET data. *J Cereb Blood Flow Metab.* 1996;16(5):834-840.

**6.** Logan J, Fowler JS, Volkow ND, Ding YS, Wang GJ, Alexoff DL. A strategy for removing the bias in the graphical analysis method. *J Cereb Blood Flow Metab.* 2001;21(3):307-320.

**7.** Logan J, Alexoff D, Fowler JS. The use of alternative forms of graphical analysis to balance bias and precision in PET images. *J Cereb Blood Flow Metab.* 2011;31(2):535-546.

**8.** Price JC, Klunk WE, Lopresti BJ, et al. Kinetic modeling of amyloid binding in humans using PET imaging and Pittsburgh Compound-B. *J Cereb Blood Flow Metab.* 2005;25(11):1528-1547.

**9.** Lopresti BJ, Klunk WE, Mathis CA, et al. Simplified quantification of Pittsburgh Compound B amyloid imaging PET studies: a comparative analysis. *J Nucl Med.* 2005;46(12):1959-1972.

**10.** Raji CA, Becker JT, Tsopelas ND, et al. Characterizing regional correlation, laterality and symmetry of amyloid deposition in mild cognitive impairment and Alzheimer's disease with Pittsburgh Compound B. *J Neurosci Methods.* 2008;172(2):277-282.

**11.** Johnson KA, Gregas M, Becker JA, et al. Imaging of amyloid burden and distribution in cerebral amyloid angiopathy. *Ann Neurol.* 2007;62(3):229-234.

**12.** Hedden T, Van Dijk KR, Becker JA, et al. Disruption of functional connectivity in clinically normal older adults harboring amyloid burden. *The Journal of neuroscience : the official journal of the Society for Neuroscience.* 2009;29(40):12686-12694.
